# Optimising the Performance of CO_2_-Cured Alkali-Activated Aluminosilicate Industrial By-Products as Precursors

**DOI:** 10.3390/ma16051923

**Published:** 2023-02-25

**Authors:** Ghandy Lamaa, David Suescum-Morales, António P. C. Duarte, Rui Vasco Silva, Jorge de Brito

**Affiliations:** 1 CERIS, Civil Engineering, Architecture and Georresources Department, Instituto Superior Técnico, Universidade de Lisboa, Av. Rovisco Pais, 1049-001 Lisbon, Portugal; 2 Área de Ingeniería de la Construcción, Edificio Leonardo da Vinci, Universidad de Córdoba, Campus de Rabanales, E-14071 Córdoba, Spain

**Keywords:** alkali-activated materials, municipal waste incinerated ashes, electric arc furnace slag, waste glass rejects, optimization, durability, construction materials

## Abstract

Three industrial aluminosilicate wastes were studied as precursors to produce alkali-activated concrete: (i) electric arc furnace slag, (ii) municipal solid waste incineration bottom ashes, and (iii) waste glass rejects. These were characterized via X-ray diffraction and fluorescence, laser particle size distribution, thermogravimetric, and Fourier-transform infrared analyses. Distinctive combinations of anhydrous sodium hydroxide and sodium silicate solution were tried by varying the Na_2_O/binder ratio (8%, 10%, 12%, 14%) and SiO_2_/Na_2_O ratio (0, 0.5, 1.0, 1.5) to find the optimum solution for maximized mechanical performance. Specimens were produced and subjected to a three-step curing process: (1) 24 h thermal curing (70 °C), (2) followed by 21 days of dry curing in a climatic chamber (~21 °C, 65% RH), and (3) ending with a 7-day carbonation curing stage (5 ± 0.2% CO_2_; 65 ± 10% RH). Compressive and flexural strength tests were performed, to ascertain the mix with the best mechanical performance. The precursors showed reasonable bonding capabilities, thus suggesting some reactivity when alkali-activated due to the presence of amorphous phases. Mixes with slag and glass showed compressive strengths of almost 40 MPa. Most mixes required a higher Na_2_O/binder ratio for maximized performance, even though, contrary to expectations, the opposite was observed for the SiO_2_/Na_2_O ratio.

## 1. Introduction

Since the early 19th century, ordinary Portland cement (OPC) has been used as the main construction binding material that gained a noteworthy reputation through its proven remarkable performance, which has been adding significant value to the construction industry since then [1]. This binding material gained relevance due to its outstanding properties that can be seen today in many designed concrete megastructures such as long-span suspension bridges, massive dams, and skyscrapers [2]. Consequently, the dependence on this construction material has never been decreasing, since as more research is made on it, its properties are evidenced, leading to an increase in the number of applications and demand [3]. Nevertheless, OPC has its disadvantages, mainly related to the environment. It has been estimated that 4270 Mt of OPC were produced during 2021, releasing around 2520 Mt of CO_2_ to the atmosphere, equivalent to the weight of around 6950 Empire State Buildings combined, wherein 0.59 t of CO_2_ is emitted per each ton of OPC produced [4,5]. The amount of CO_2_ released by the cement industry in 2021, accounted for 6–8% of the total CO_2_ emissions released globally by all industries in that same year, gaining a spot in the top 10 CO_2_-generating industries [6]. Moreover, in the last decade, there have been several attempts to cut CO_2_ emissions to lessen the global warming consequences on the Planet [7]. Since the cement industry is responsible for a major share of these emissions, reducing them is considered to influence participation in the global warming mitigation program.

Reducing the emissions resulting from the production of OPC has been tried in many techniques deployed in past years. Some of these techniques were technological-based and focused on improving the cement production phases to be more energy efficient and release less CO_2_. One example includes the possibility of reducing the CO_2_ emissions at a cement plant through the optimization of the cement calciner’s geometry and operating conditions [8]. Nevertheless, most studies have focused on reducing the OPC’s environmental impacts by replacing it with supplementary cementitious materials (SCMs) such as blast-furnace slag (BFS), fly ash (FA), and silica fume (SF), which have been widely used as OPC partial replacements [9,10,11,12].

Partially replacing OPC with SCM has shown considerably reduced environmental impacts. For this reason, there have been several studies on the use of aluminosilicate-rich binders to fully replace OPC in the production of alkali-activated materials (AAMs) [13,14,15]. Unlike the hydration mechanism of OPC, the aforementioned SCMs, hereon presented as precursors, are activated using an alkaline solution. The use of FA and BFS as sole and/or blended precursors in alkali-activated concrete mixes has been widely investigated with interesting results reported in the literature [16,17,18,19,20,21,22,23,24,25,26]. This has led to a revolutionary eco-friendly concept since both are considered industrial by-products and exist in bulk quantities enough to cover part of the market demand. However, FA and BFS are now facing shortages in availability, due to the use of renewable energy sources for energy production and the use of alternative more eco-efficient processes [27,28,29]. The source of FA is the coal industry, and this product is generated at coal-fired power plants. However, burning coal does not only just produce power but also generates thousands of harmful chemicals that are consistently being released to the atmosphere. For this reason, this type of power plant is being shut down. In Portugal, for instance, the last two remaining coal-fired power plants were shut down in 2021 [30]. Similarly, the production of BFS will diminish significantly in the near future, as the steel industry is more focused on switching to steel recycling using electric arc furnaces (a more energy-efficient process), rather than producing new steel from iron ore.

New aluminosilicate industrial waste materials have been investigated recently considering their chemistry and binding capabilities. The binding capability of municipal solid waste incinerator bottom ashes (MIBAs) and electric arc furnace slag (EAFS) as sole precursors in alkali-activated materials was demonstrated in some studies [28,31,32,33,34,35]. Mortar and concrete containing these binders generally show low mechanical and durability-related performances [28,32], mainly due to the lower amount of amorphous content in the precursors and the porous microstructural profile of the hardened materials [32,34]. Research on this matter evolved with the application of accelerated carbonation as a performance-enhancing curing method. Some authors [36,37] began investigating the influence of a CO_2_-based curing method on the properties of alkali-activated concrete and have observed notable enhancements in the porosity and microstructure, as well as in the mechanical performance [36,38,39,40,41,42,43,44,45,46,47,48,49].

Given the discrepancy in the chemical composition of the precursors used for alkali activation, specific formulations are needed for optimal performance. The alkaline solution is prepared by mixing several compounds (e.g., NaOH, KOH, and “water glass”), the optimal concentration of which will lead to improvements in the hydration process in binding performance, thus leading to enhancements in several properties [50]. Kassim et al. [36] optimised the alkaline solution for the EAFS precursor, and the optimal alkaline solution showed significant enhancements in mechanical performance. This study as well as others made on MIBA proved the efficiency of this technique in improving the performance of alkali-activated binders [46]. However, those studies [36,46] investigated a small range of ratios of the alkaline activators and only for mortar production, thereby calling for the need for further investigation on the matter, specifically for fit-for-industry construction materials such as non-structural precast concrete elements (e.g., pavement blocks). Only then the true binding performance of these precursors can be ascertained, and their readiness for the market can be confirmed. Therefore, in this study, FA, MIBA, EAFS, and waste glass rejects (WGR) were used as precursors to produce alkali-activated small-scale specimens that are representative of conventional concrete pavement blocks. The choice of industrial by-products studied was based on various criteria including availability in Portugal, the region where the study was conducted, sustainability, cost-effectiveness, and technical feasibility (i.e., activation potential). The motivation behind the present work came in response to the growing demand for sustainable and environmentally friendly materials, such as the use of waste materials as binder alternatives such as ordinary Portland cement and FA, which would otherwise keep consuming natural resources and contributing to the global emissions and environmental pollution.

The study focused on maximizing the performance of these binders by optimising their alkaline solutions, where 14 different formulations were prepared per binder. Afterwards, the specimens were subjected to a three-stage curing process and subsequently tested for flexural and compressive strengths to ascertain the maximized mechanical performance for an optimal alkaline solution for any given binder.

## 2. Materials and Methods

### 2.1. Binders

In this study, CEM I 42.5 R was used as the reference binder, in accordance with EN 197-1 [51]. The reference binder for the alkali-activated materials was FA, supplied by Energias de Portugal (EDP), from the Sines power plant in Alentejo, Portugal. Since this FA particles met the size requirements (average particle size below 45 μm), no prior preparations were required. MIBA was obtained from the Valorsul waste-to-energy power plant in São João da Talha, Portugal. It required previous preparation before it was used as a precursor, which included the removal of large contaminants (e.g., paper, plastic, and metals), drying at 105 °C, and grinding it to meet the particles’ size distribution typically required for binders. EAFS, a by-product of the steel recycling industry, was obtained from Siderurgia Nacional de Portugal and supplied by HARSCO in Portugal. Similar to MIBA, EAFS required size reduction since it presented an extensive and coarse particle size distribution. Furthermore, the WGR used in this experimental campaign was supplied by CascoVidro, in Marinha Grande, Portugal. WGR corresponds to the rejects resulting from the infrared contaminant separation stage from curbside-separated glass packaging waste. These contaminants (e.g., aluminum caps, paper labels, ceramic dishware, “Pyrex”, along with other minor components) are a result of the incorrect separation at households and are the reason why waste glass cannot be recycled into new consumer glass packaging products without a separation stage. However, the rejects from this stage are mostly comprised of highly reactive soda-lime-silica glass and thus compatible with alkali activation. WGR presents an extensive particle size distribution thereby requiring size reduction via milling.

### 2.2. Aggregates

In this campaign, two types of aggregates were used: siliceous sand and calcareous sand gravel. As for the siliceous sands, 0/4 coarse sand and 0/2 fine sand were used in accordance with standard EN 12620 [52]. With respect to sand gravel, which was calcareous, it had a particle size between 2 mm and 5.6 mm. Before use, all aggregates were fully dried at 105 °C.

### 2.3. Alkaline Activator

The alkaline activator used was a liquid solution prepared using sodium hydroxide, sodium silicate solution, and water. Reactive grade sodium hydroxide pellets in the solid state with 98% purity, and 2.13 g/mL of density were used. In addition, a reactive grade sodium metasilicate solution was used containing: a silicon oxide content of (SiO_2_) of 26.4 ± 1.5%, a sodium oxide (Na_2_O) content of 8 ± 0.6%, a SiO_2_/Na_2_O ratio of 3.3 ± 0.1, water content of 65.6 ± 2%, and a density of 1.355 g/mL. The sodium hydroxide was dissolved in potable tap water, which was provided by Empresa Portuguesa de Águas Livres (EPAL), Portugal. The water used complied with Directive 98/83/CE [53].

### 2.4. Water Reducing Admixture

In this study, SikaPlast-717, which is a naphthalene-based superplasticizer, was used as a water-reducing admixture (WRA). It consists of synthetic organic water-based dispersants with a density of 1.21 ± 0.03 g/mL and a pH of 10 ± 1.

### 2.5. Setting Time Retarder

A setting time retarder was required and used in this study. Borax decahydrate (37%) was dissolved in the alkaline solution for all mixes.

### 2.6. Mix Design

In this study, 16 mixes were prepared per precursor, each having a unique combination of Na_2_O/precursor and SiO_2_/Na_2_O ratios. The Na_2_O/precursor percentages chosen were 8%, 10%, 12%, and 14%, while the SiO_2_/Na_2_O ratios were 0, 0.5, 1.0, and 1.5 (Table 1). To achieve the required consistence (slump flow of 105 ± 5 mm), the cement-based mix required 1.5% WRA by mass of the binder. In addition, an amount of 2% WRA by binder mass was required in the WGR mixes. Moreover, no WRA was required for FA, MIBA, and EAFS, since their mixes’ consistence was achieved without it. From each family mix, three specimens were produced. A water-to-binder ratio (*w*/*b*) of 0.35 was used for all mixes. To prevent flash setting, 4% of setting retarder by mass of binder was added and dissolved in the alkaline solution of all alkali-activated mixes as recommended in other studies [13,54]. The OPC and AAMs mixes composition (in kg/m^3^) are shown in Table 2.

### 2.7. Production Method and Curing Regime

As mentioned, the specimens prepared were representative of conventional concrete pavement blocks, but with the dimensions of standard mortar specimens. The same formulation was used, and a comparable compaction method was implemented. The relatively smaller size of specimens allowed improved compatibility with existing testing equipment. Therefore, the preparation and production of the mixes were carried out in compliance with EN 196-1 [55], albeit with some modifications, mainly to the mixing procedures. Firstly, the alkaline solution was prepared by gradually dissolving NaOH in a pre-measured additional amount of water, followed by the addition and dissolution of the sodium silicate solution and the setting time retarder. The solution was stirred until complete dissolution of all components was ensured. The full amount of sand–gravel was added first with two-thirds of the prepared solution, then mixed at normal speed for 4 min. Afterwards, the fine and coarse sands were added to the bowl and mixed for 2 more minutes. Finally, the binder and the remaining one-third of the solution (plus the WRA amount for OPC and WGR mixes) were added and mixed for 4 additional minutes. The release agent used for the 40 × 40 × 160 mm^3^ three-gang steel molds was Petromold-F given its higher release effectiveness for more alkaline mixes. The mix was then molded, compacted, and covered with a plastic film before it was transported to a thermal curing chamber for 24 h except for the reference OPC mix. For this binder, at the early stages of curing, the specimens were sprayed with water to make the curing process compatible with that followed by the industry. Following this stage, the specimens were demolded and moved to a dry curing chamber (~65% RH and ~23 °C) for 21 days. Finally, all specimens were placed in a carbonation curing chamber (~65% RH, ~23 °C, and 5% CO_2_) for 7 days. The total curing duration for all specimens was 28 days. The curing stages and conditions are presented in Table 3.

### 2.8. Characterisation and Testing Methods

The density of all binders, at atmospheric pressure, was measured with a gas pycnometer (MICROTRAC BELPycno Ver 1.14 L) using helium gas.

The chemical composition of raw materials was determined via the X-ray fluorescence spectrometry (XRF) method, using a ZSX PRIMUS IV (Rigaku) with a power of 4 kW.

The X-ray diffraction method (XRD) applied was performed using a Bruker D8 Discover A25 instrument with Cukα (λ = 1.54050 A, 40 kV and 30 mA). The diffraction patterns were obtained with a goniometric scan from 10° to 70° (2θ) at the speed of 0.016 2θ·s^−1^. The diffractogram peaks of the crystalline phases were compared with those of the JCPDS library [56].

The particle size distribution (PSD) was obtained with a Mastersizer S. Analyser (Malvern Instruments) using ethanol as a dispersant, sonicated for 5 min before analysis.

Moreover, the thermogravimetric analysis (TGA) and differential thermal analysis (DTA) were carried out in a Setaram Setys Evolution 16/18 apparatus, using alumina crucibles under airflow and argon. The heating rate was 5 °C.min^−1^, and the temperature range was between 20 °C and 1000 °C.

Furthermore, the morphology and composition of WGR were obtained using scanning electron microscopy (SEM), energy-dispersive X-ray spectroscopy (EDS), and backscattered electron (BSE) imaging with a JEOL JSM 7800 F. The sample was dusted on a carbon tape. A gold sputtering was used to improve the conductivity of the samples.

The consistency of the fresh mixes was tested in accordance with EN 1015-3 [57]. After curing, the specimens were tested for flexural and compressive strength in accordance with EN 1015-11 [58]. The loading rate applied for the flexural and compressive strength tests had a constant value of 30 N/s and 300 N/s, respectively.

## 3. Results

### 3.1. Materials Characterisation

#### 3.1.1. Binder Density

The apparent skeletal density of OPC, FA, MIBA, EAFS, and WGR were 3115 kg/m^3^, 2431 kg/m^3^, 2704 kg/m^3^, 3770 kg/m^3^ and 2531 kg/m^3^, respectively.

#### 3.1.2. X-ray Fluorescence

The chemical compositions of the raw materials obtained from XRF are presented in Table 4. The sum of oxides Al_2_O_3_ + Fe_2_O_3_ + SiO_2_ corresponds to 26.4%, 88.7%, 64.3%, 56.4%, and 73.1% of the composition of OPC, FA, MIBA, EAFS, and WGR, respectively. Concerning EAFS, it contains a high amount of iron that gives it magnetic properties. With the use of neodymium magnets, the alkali-activated EAFS precursor was proven to be strongly magnetic [59]. High amounts of Fe(III) can lead to the formation of iron oxide (Fe_2_O_3_) precipitates. These can decrease the overall reactivity of the system, reducing the amount of available reactive species and limiting the degree of polymerization and network formation that can occur. This can lead to a decrease in the overall strength and durability of the AAM. The XRF results of WGR show that it is mainly composed of silicon dioxide (~71% SiO_2_), followed by Na_2_O, CaO, and MgO. The first three are typically present in conventional soda-lime silicate glass used in containers for human consumption. Other chemical components, such as Fe and Al, were also identified. According to ASTM C618 [60], WGR can be identified as a standard pozzolan since the sum of oxides (SiO_2_ + Al_2_O_3_ + Fe_2_O_3_) exceeds 70% of its total constituents. Others have also verified its high pozzolanicity [61,62,63].

**Figure 1:**
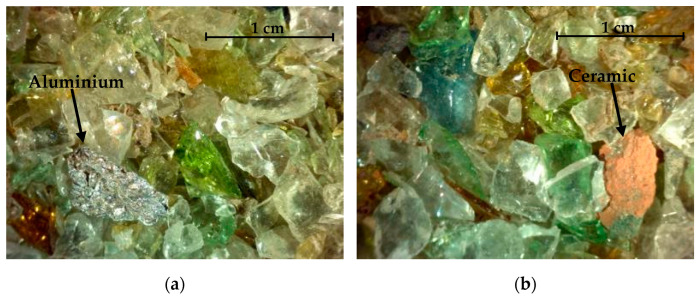
Magnified images of a WGR sample showing the presence of (**a**) aluminum and (**b**) ceramic particles in parallel with glass particles.

In addition to the previous analysis, an image magnifier device was used to ascertain the main morphological characteristics of a washed sample of WGR before milling (Figure 1). The WGR contains a considerable amount of glass particles. This suggests that the infrared separation process, initially designed to remove contaminants from waste glass, also ends up removing much of the glass particles. Naturally, the presence of other constituents such as aluminum and ceramics was also observed, which are mostly derived from bottle caps and dishware.

#### 3.1.3. X-ray Diffraction

**Figure 2:**
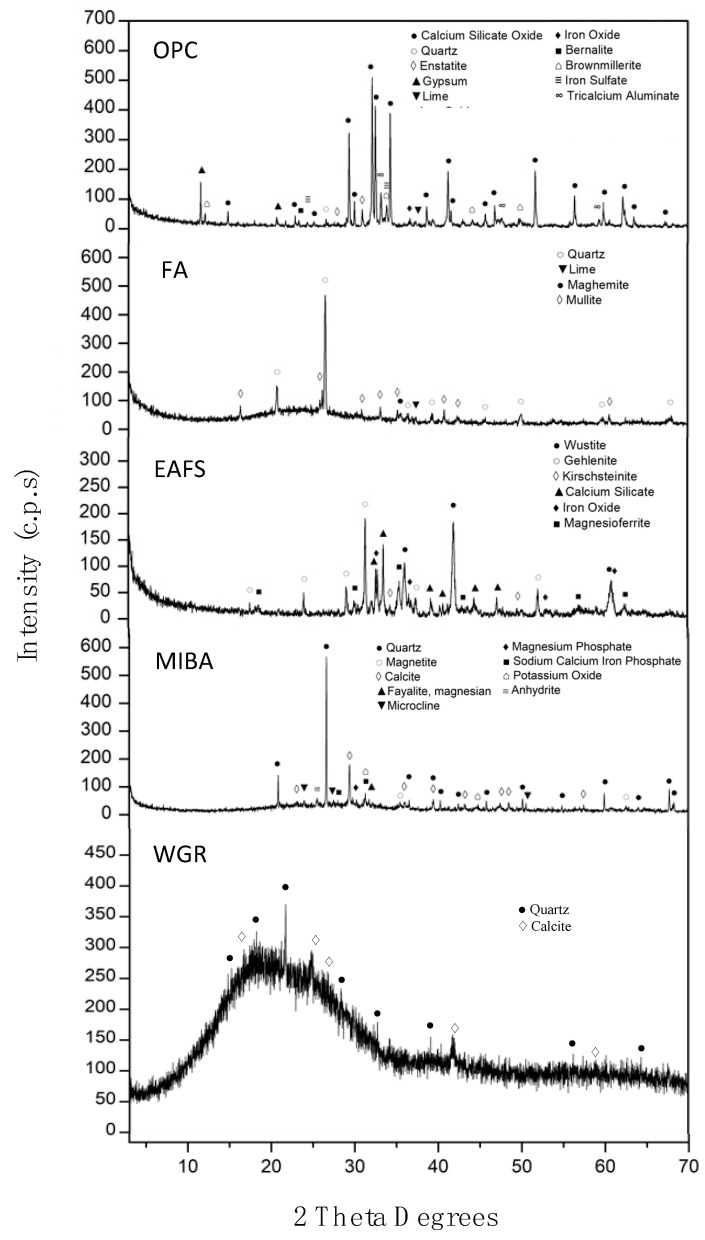
XRD analysis of OPC, FA, EAFS, MIBA, and WGR.

Figure 2 presents the XRD results of OPC, FA, MIBA, and EAFS. The XRD spectrum of OPC showed crystalline peaks for calcium silicate oxides (Ca_2_SiO_4_) and gypsum (CaSO_4_·2H_2_O). The main crystalline phases observed for FA were quartz (SiO_2_), lime (CaO), maghemite (Fe^3+^_2_O_3_), and mullite (3Al_2_O_3_·2SiO_2_). Some studies have reported a similar observation [64,65]. In addition, a broad reflexion peak can be seen at 15–35 2θ°, which can indicate the presence of a considerable amount of amorphous phases. Concerning MIBA’s XRD spectrum, the crystalline phases observed were quartz (SiO_2_), calcite (CaCO_3_), magnetite (Fe^2+^Fe^3+^_2_O_4_), fayalite ((Fe^2+^)_2_SiO_4_), magnesium carbonate (MgCO_3_), microcline (KAlSi_3_O_8_), along with other minor minerals. The presence of amorphous phases (reflexion peak ranging between 20° and 40° 2θ) can be observed, which is mostly related to the presence of waste glass fraction; however, it exists at a lower degree when compared to FA. Regarding EAFS, the three main crystalline phases noticed were wustite (FeO), gehlenite (Ca_2_Al_2_SiO_7_), and dicalcium silicate (Ca_2_SiO_4_), along with other minor phases such as magnesioferrite. WGR shows a diffractogram with an essentially amorphous structure, also observed in other studies [66,67]. This further confirms that almost all of the particles present in WGR are from consumer glass containers and that it may show high reactivity when alkali-activated. Within this amorphous structure, the two main crystalline phases found were the quartz phase (SiO_2_) and the calcite phase (CaCO_3_). Other minor phases may be present as well, but the amorphous nature of WGR did not allow them to be clearly defined.

#### 3.1.4. Particle Size Distribution

**Figure 3:**
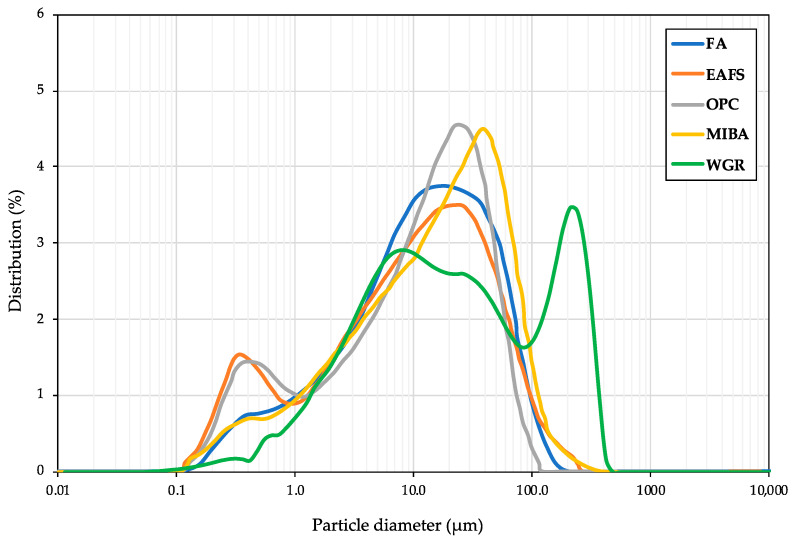
Particle size distribution for all precursors.

Figure 3 shows the PSD for OPC, FA, MIBA, EAFS, and WGR. The samples of OPC, FA, and EAFS indicated a similar bimodal distribution curve. OPC and EAFS peaked at 25 μm; however, a narrower peak was seen for OPC compared to EAFS. In additon, both binders showed a smaller peak at 0.35 μm, representative of a noteworthy presence of very fine particles. The FA’s distribution followed a similar trend; however, a wider distribution was noticed around 20 μm (peak). A small peak was seen at 0.35 μm for FA as well. Furthermore, MIBA showed a narrow peak at 39 μm with a small one at 0.35 μm. Concerning WGR, its PSD showed a slightly different profile in comparison with the other binders, despite having had the same milling procedure.

In Figure 3, the distribution of particles peaked at 7.58 μm with a narrow top peak at 209 μm. A smaller peak was observed at 0.47 μm. The distribution of WGR is similar to the one reported by Jiang et al. [68]. Zhang et al. [61] studied the effects of WGR particle size on its chemical reactivity with the alkaline solution. The authors of that study concluded that, when the WGR particles’ diameter exceeded 300 µm, the mix showed low chemical reactivity and hardly had any reaction with the alkaline solution. However, as expected, the smaller the particle size of WGR, the higher the chemical reactivity, and consequently, the highest mechanical performance achieved.

**Figure 4:**
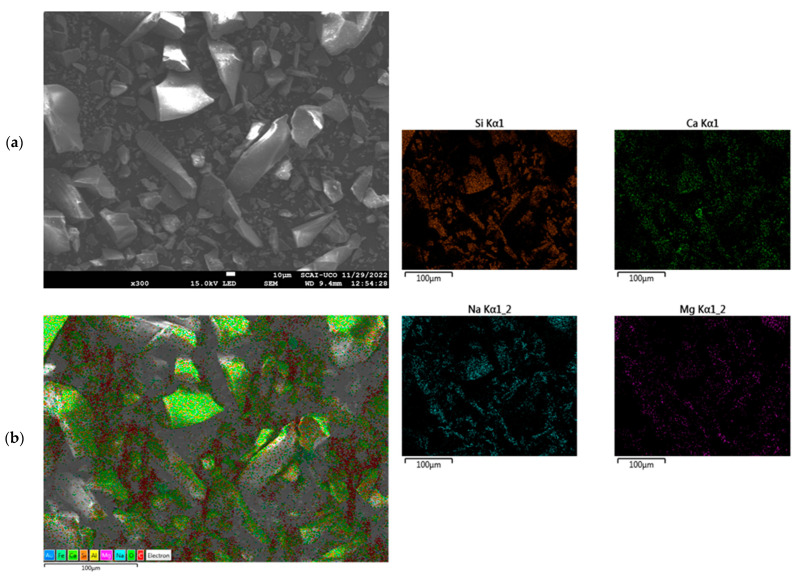
SEM images (**a**) and mapping (**b**) for WGR.

Previous studies by the authors have interpreted the particles’ morphology of FA, MIBA, and EAFS using SEM images and mapping [36,69]. The SEM analysis conducted by Kassim et al. [36] revealed that EAFS particles are irregular in shape and showed high angularity. Moreover, the particle morphology of FA and MIBA were studied by Suescum-Morales et al. [69] indicating spherical particles shape for FA. MIBA, however, showed angular edges with a porous microstructure. The authors suggested that a larger amount of water could be needed for a given workability level, in the case of MIBA. The particles’ morphology and composition of WGR were investigated using SEM and mapping, respectively. Figure 4 shows that the majority of the particles present high angularity and irregular shape. This observation complies with the findings of other studies [67,68]. This high angularity of WGR could lead to a higher water requirement of mixes and loss in workability [70], yet can also be responsible for an enhanced interlocking of particles, thereby increasing the mechanical strength. Furthermore, mapping revealed the presence of the main chemical components already observed in the XRF analysis (Si, Ca, Na, and Mg in Figure 4). The presence of other components such as Fe and Al was also noticed though to a lesser extent.

#### 3.1.5. Thermogravimetric Analysis

**Figure 5:**
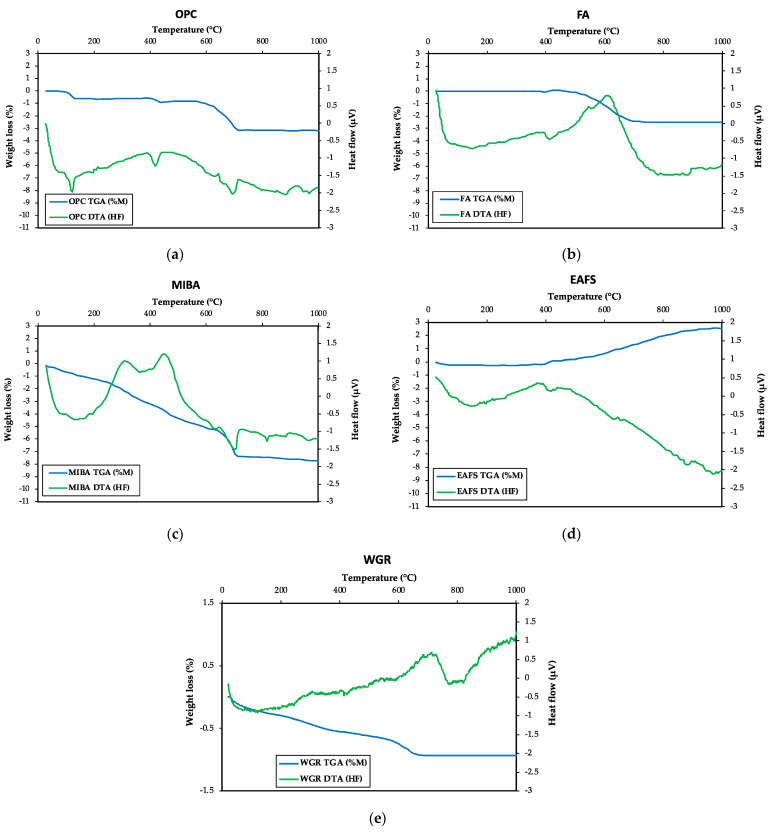
TGA-DTA analysis of (**a**) OPC, (**b**) FA, (**c**) MIBA, (**d**) EAFS, and (**e**) WGR (inert argon atmosphere).

Figure 5 shows the TGA/DTA results of OPC, FA, MIBA, EAFS, and WGR. For OPC (Figure 5a), the profile suggests that the hydrated calcium sulphate (gypsum) that appeared in the XRD spectrum transformed into hemihydrated calcium sulphate at 110 °C. At around 200 °C, it is possible that unhydrated calcium sulphate formed. At 420 °C, around 0.4% of portlandite became depleted due to dehydroxylation. This low amount of lost portlandite reflects its low hydration rate and that it is well stored. Moreover, from 600 °C onwards, the compounds formed by calcium carbonate were decarbonated.

The TGA results of FA indicate two phenomena (Figure 5b). The first is the mass loss starting from 400 °C, which may correspond to the unburnt coal particles present in the FA (exothermic peak noticed in the DTA profile). From ~600 °C onwards, the endothermic peak suggests that there may have been a slight mass loss of CO_2_ of some carbonates.

Concerning MIBA, mass losses were observed in four phases (Figure 5c). The initial mass loss may have been due to the dehydration of the sample (up to around 105 °C). After that, the losses could have been caused by the decomposition of phosphates as well as organic matter. Moreover, the mass loss at ~650 °C may be associated with the decarbonation of calcite and magnesite.

Unlike other binders, a gain in mass was observed with the EAFS sample (Figure 5d). The X-ray diffractogram of the sample after the TGA analysis phase (heated up to 1000 °C) indicated this steady increase in mass, mainly at ~400 °C and ~620 °C, could be explained by the oxidation of the wustite, first in magnetite and then in hematite.

For WGR (Figure 5e), despite the relatively small amount of mass loss, several stages were observed: (i) loss of moisture from room temperature to 105 °C [71]; (ii) possible loss of organic matter (e.g., food residue) between 105 °C and 380 °C due to the exothermic peaks found [72]; (iii) endothermic peak was observed in the DTA graph at around 420 °C caused by the dehydroxylation of trace amounts of Ca(OH)_2_ [71,73]; (iv) loss in mass due to decarbonation of the calcite phase found in X-ray diffractogram (Figure 2) after ~600 °C [29,69].

#### 3.1.6. Fourier-Transform Infrared Analyses

**Figure 6:**
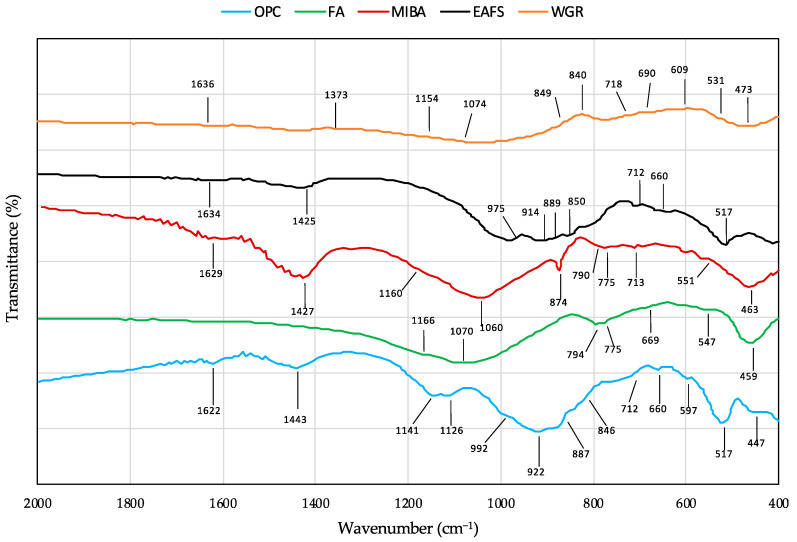
FTIR spectra of OPC, FA, MIBA, EAFS, and WGR.

The Fourier-transform infrared analysis (FTIR) for OPC, FA, MIBA, EAFS, and WGR is shown in Figure 6. Starting with the OPC spectrum, the band observed at 1622 cm^−1^ was associated with the O–H bending of water [74]. Additionally, the band at 1443 cm^−1^ was linked to an asymmetric stretch of CO_3_ in calcite from the carbonation of CaO (lime). The bands at 1141 cm^−1^ and 1126 cm^−1^ were associated with the symmetric and antisymmetric stretch vibration modes of tetrahedral SiO_4_ groups in gypsum, respectively. Moreover, the bands at 992 cm^−1^ and 846 cm^−1^ were related to the stretching of Si–O bonds within the tetrahedral SiO_4_ groups in C_2_S. Furthermore, the bands at 887 cm^−1^ and 922 cm^−1^ were related to the symmetric and antisymmetric stretching of Si–O bonds, respectively, within the tetrahedral SiO_4_ groups in C_3_S. The band at 712 cm^−1^ was related to the AlO_4_-tethahedral groups in C_3_A. Additionally, the band at 660 cm^−1^ was associated with FeO_4_-tethahedral groups in C_4_AF. Furthermore, the bands at 660 cm^−1^ and 597 cm^−1^ were linked to the antisymmetric bending vibrations of SiO_4_ in gypsum. Moreover, the two bands at 517 cm^−1^ and 447 cm^−1^ were due to symmetric and antisymmetric bending of the O–Si–O bonds in C_3_S, respectively [75].

In the FA spectrum, the bands appearing around 1166 cm^−1^ and 1070 cm^−1^ correspond to the asymmetric stretching vibrations of Si–O–Si associated with quartz and mullite [76,77]. In addition, the bands at 794 cm^−1^ and 775 cm^−1^ correspond to symmetric stretching vibrations of Si–O–Si bonds associated with the characteristic doublet of quartz [77]. Moreover, the bands observed at 669 cm^−1^ and 547 cm^−1^ were associated with the O–Al–O vibration [74]. The band presented at 547 cm^−1^ was linked with the O–Al–O vibration corresponding to the octahedral aluminum present in mullite. Additionally, the band appearing at 459 cm^−1^ was associated with either O–Si–O or O–Al–O bonds bending vibration [74].

Regarding the EAFS spectrum, the band at 1634 cm^−1^ was associated with the O–H bending of water, while the band at 1425 cm^−1^ was related to the asymmetric stretching of CO_3_^2−^ [74]. The bands 975 cm^−1^, 914 cm^−1^, 889 cm^−1^, and 850 cm^−1^ were associated with the stretching Si–O bonds within the tetrahedral SiO_4_ groups [75,78]. The band shown at 712 cm^−1^ was due to AlO_4_-tethahedral groups in C_3_A, while the band 660 cm^−1^ was associated with FeO_4_-tethahedral groups. Moreover, the band at 517 cm^−1^ was linked to the symmetric and antisymmetric bending of the O–Si–O bonds [75].

Regarding the MIBA spectrum, the band at 1629 cm^−1^ was associated with the vibrations of O–H bonds (water). In addition, the absorbance located at 1427 cm^−1^, 874 cm^−1^, and 713 cm^−1^ was attributed to the vibrations of CO_3_^2-^ [79]. Moreover, the bands appearing at 1160 cm^−1^ and 1060 cm^−1^ correspond to the asymmetric stretching vibrations of Si–O–Si that are associated with quartz. Furthermore, the characteristic doublet of quartz at 790 cm^−1^ and 775 cm^−1^ corresponds to the symmetric stretching vibrations of Si–O–Si bonds. In addition, the bands at 551 cm^−1^ and 463 cm^−1^ were attributed to the Si–O vibrations (quartz) [77].

Regarding the WGR spectrum, the bands at 1074 cm^−1^ and 1154 cm^−1^ may be associated with the Si-O-Si stretching vibration. The band at 1636 cm^−1^ was associated with the vibrations of O-H bonds (water). The absorbance located at 1373 cm^−1^ and 849 cm^−1^ was attributed to the vibrations of CO_3_^2-^. This is in accordance with the calcite found in the XRD results (Figure 2). Those located at 473 cm^−1^, 531 cm^−1^, 609 cm^−1^, 690 cm^−1^, and 718 cm^−1^ can be associated with the bending vibration of Si-O.

### 3.2. Fresh-State Performance

The consistency of all fresh mixes was tested in accordance with EN 1015-3 [57]. The OPC mix was prepared by considering a w/b ratio of 0.35 and 1.5% of superplasticizer. The AAM mixes were prepared following the same w/b ratio as that of the OPC mix, though with different WRA content (see Section 2.6). For the OPC mix, the flow table results indicated an average value of 108.5 mm. This value was slightly higher than most of the results observed for AAMs with the other binders, except for FA, where considerably high values were recorded. Moreover, the consistency test for AAM mixes showed comparable performances with some families. As shown in Table 5, formulations N10S0.5, N12S0.5, and N14S0.5 consistently resulted in considerably stiff mixes, regardless of the precursor. Additionally, this phenomenon also occurred for some N8S0.5 mixes.

Generally, the flow value of AAMs decreases with the increase in Na_2_O/precursor and SiO_2_/Na_2_O ratio. These lead to an increase in viscosity and, due to the higher content of SiO_2_, supersaturation is achieved more rapidly leading to flash setting [80,81]. It is possible that the aforementioned ratios create the ideal conditions for more rapid polymerization to occur; additional research on this matter is needed. Others showed that higher flow values can be witnessed with the increase in SiO_2_/Na_2_O ratio in the alkaline solution [82]. The latter study’s conclusion is similar to what is shown in Table 5, where higher Na_2_SiO_3_ content increases the workability of the mixes. The influence of the NaOH concentration on the consistency of the mixes did not follow any trend, unlike what was reported by Li et al. [83], where the decrease in NaOH concentration led to an increase in workability. Furthermore, N12S0 and N14S0 with only NaOH as an alkaline activator showed better performance than the mixes with sodium silicate solution. This was also evidenced by Laskar and Talukdar [84] by concluding that better workability can be achieved when only NaOH is used as the alkaline activator, compared to solutions with high SiO_2_/Na_2_O ratios. Moreover, it is difficult to compare the influence of the changing proportions of the alkaline solution constituents on the working performance of different activated precursors, since each has its unique chemistry that allows different reactions with the alkaline solution [85].

### 3.3. Hardened-State Performance

#### 3.3.1. Compressive Strength

**Figure 7:**
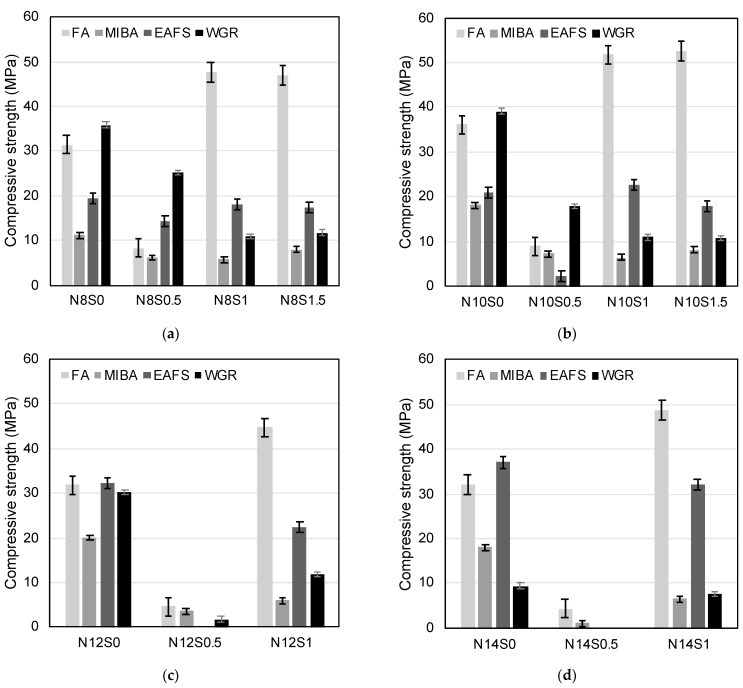
Compressive strength values for mixes with FA, MIBA, EAFS, and WGR with Na_2_O/binder ratios of (**a**) 8%, (**b**) 10%, (**c**) 12%, and (**d**) 14%, respectively.

For the OPC mix, the average compressive strength value reported was 56.7 MPa (±3.5 MPa standard deviation). The average compressive strength values for each family of the four alkali-activated binders are presented in Figure 7. The best performance with FA was achieved at N10S1.5 and N10S1 mixes, with average compressive strength values of 52.5 MPa and 51.9 MPa, respectively (Figure 7b). For MIBA, the highest values were 20.0 MPa for the N12S0 mix and 18.1 MPa for both N10S0 and N14S0 mixes (Figure 7b–d). Similarly, EAFS peak values were seen for N14S0 mix with 37.0 MPa; the N12S0 mix showed a compressive strength of 32.4 MPa (Figure 7c,d). Moreover, the highest compressive strength values for WGR were reported for N10S0, N8S0, and N12S0 mixes with values of 39.1 MPa, 35.9 MPa, and 30.3 MPa, respectively (Figure 7a–c).

**Figure 8:**
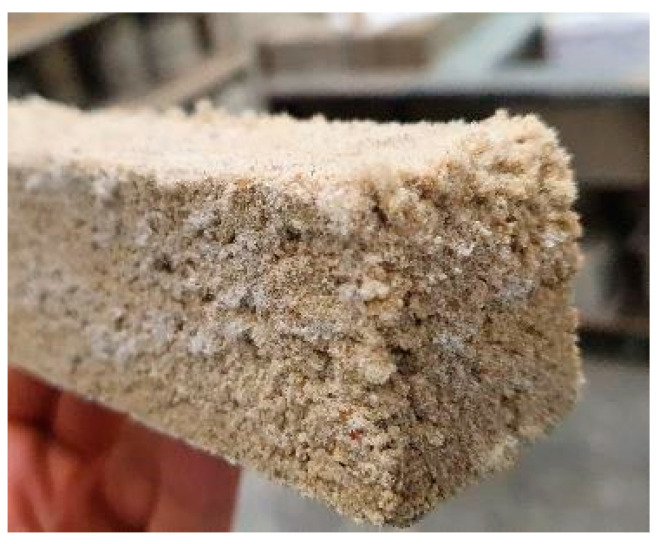
Efflorescence caused by sodium carbonate precipitation on the surface of N14S0 WGR specimen.

Although the best performance was generally attained by specimens with higher Na_2_O content, the opposite was seen in WGR-containing mixes; the corresponding N14S0 mix showed a low value of ~9.5 MPa, whereas those with FA, MIBA, and EAFS presented values of ~32 MPa, ~18 MPa and ~37 MPa, respectively. The concentration of Na^+^ was much higher (both from the activator as well as the precursor itself) than needed for the WGR N14S0 mix, where notable efflorescence was noticed (Figure 8). This phenomenon occurs when the Na^+^ ions migrate to the surface of the specimens, resulting in the precipitation of sodium carbonates [36]. The high concentrations of sodium hydroxide and sodium silicate are known to affect the development of compressive strength of alkali-activated mixes [36,86]. Vafaei and Allahverdi [87] investigated the tendency of alkali-activated waste glass powder towards efflorescence formation. The study concluded that the tendency is dependent on the concentration of free alkali in the cured alkali-activated concrete specimens. The high free-alkali concentration increases the tendency of alkali to migrate to the surface of the sample, consequently forming efflorescence caused by the weak binding of Na^+^ ions [88].

**Figure 9:**
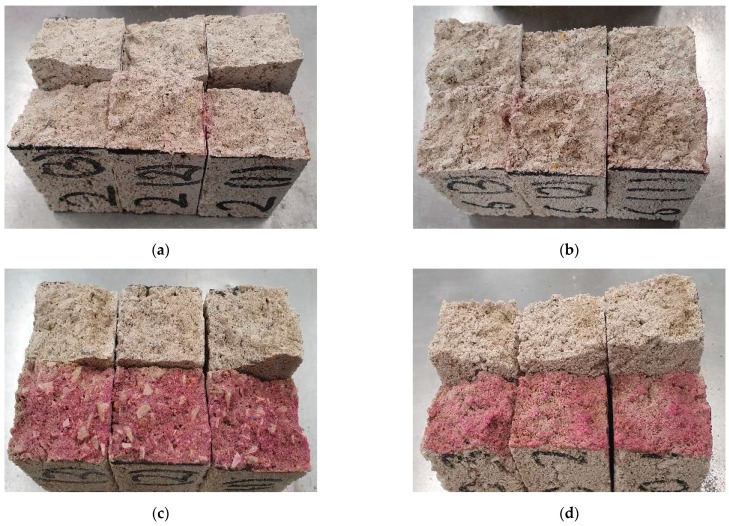
The change in color by spraying phenolphthalein solution pH indicator on (**a**) N8S0.5, (**b**) N10S0.5, (**c**) N12S0.5, and (**d**) N14S0.5 WGR mixes.

Concerning the lowest-performing mixes, generally, these corresponded to those with a SiO_2_/Na_2_O ratio of 0.5 (N8S0.5, N10S0.5, N12S0.5, and N14S0.5, except for WGR that showed intermediate values at both N8S0.5 and N10S0.5 mixes—25.1 MPa and 17.8 MPa, respectively). The mixes previously presented significantly lower workability likely due to the activator’s fast polymerization, thereby resulting in a flash setting. Naturally, this prevented optimum compaction of the mixes inside the molds, leading to more porous specimens. In spite of the lower workability of WGR mixes at the same ratio, this binder still showed relatively high cohesiveness for N8S0.5 and N10S0.5 mixes in comparison with FA, MIBA, and EAFS counterparts. One potential explanation for this is the formation of CaCO_3_ caused by the accelerated carbonation curing, which densified the microstructure by filling the internal pores created by the dry mix [28]. This can be seen in Figure 9, which presents the N8S0.5 and N10S0.5 mixes of all binders after spraying a phenolphthalein solution. The pH indicator resulted in a pinkish hue for FA, MIBA, and EAFS, unlike the WGR mixes for which nothing was shown, indicating that the N8S0.5 and N10S0.5 mixes of WGR were possibly fully carbonated and showed a greater extent of reactions into N-A-S-H and C-A-S-H.

All precursors with a formulation of N12S0.5 and N14S0.5 showed significantly low cohesiveness. After having applied the phenolphthalein solution, the surfaces showed a pinkish hue. Generally, this indicates a low carbonation depth carbonation. However, this technique, which is normally used to measure the carbonation depth of OPC concrete, is not as accurate as when used on alkali-activated materials. Apart from observing a pH decline with ensuing carbonation, it also decreases with the ongoing chemical reactions between the precursor and the alkaline solution; the OH^-^ ion in the pore solution, originally from the NaOH, is progressively embedded in the materials’ microstructure, namely in the formation of N-(C)-A-S-H phases. Therefore, the phenolphthalein solution pH indicator is not valid in establishing the front of carbonation in AAMs, but it can be used to ascertain the extent of OH^-^ taken up by reaction products and thus an indicator of the material’s performance.

Previous studies have demonstrated the positive impact of an accelerated carbonation curing stage on the strength development of alkali-activated concretes, by comparing uncarbonated specimens with carbonated ones with the same mix design [89]. During this stage, Ca-bearing phases become decalcified with ongoing exposure to a CO_2_-rich environment, leading to the formation of CaCO_3_ and amorphous Si–O structures [90]. Consequently, microstructural densification occurs leading to enhancement in mechanical performance including compressive strength [28,38,39,40,41,42,43,44]. As expected by the authors’ previous experience on the matter, FA mixes show greater enhancements in performance when compared to MIBA, EAFS, and WGR, as it contains more reactive amorphous aluminosilicate phases resulting in the creation and growth of the amount of N–A–S–H [89,91]. In addition, the FA’s specific morphology allows for a more enhanced compaction of the material, creating a denser microstructure. Nevertheless, it is worth noting that, despite the significant enhancement in performance, only a limited amount of CaCO_3_ formed in the accelerated carbonation curing stage since type F fly ash was used. It may mostly be related to the carbonation of Na^+^ in the pores’ solution, leading to the precipitation of sodium carbonate and thus densification of otherwise empty spaces.

#### 3.3.2. Flexural Strength

**Figure 10:**
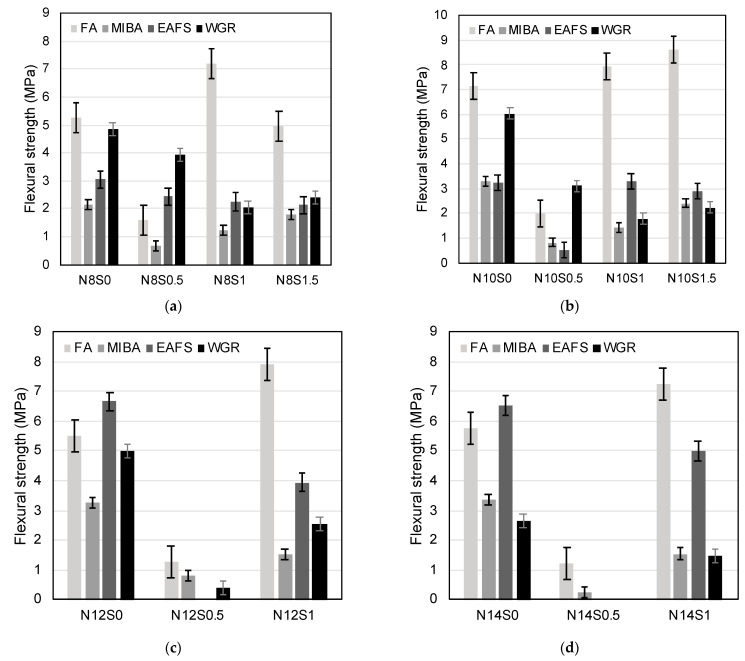
Flexural strength values for mixes with FA, MIBA, EAFS, and WGR with Na_2_O/binder ratios of (**a**) 8%, (**b**) 10%, (**c**) 12%, and (**d**) 14%, respectively.

The flexural strength test was carried out in accordance with EN 1015-11 [58]. For the OPC reference mix, the average flexural strength value reported was 6.7 MPa (±0.14 MPa of standard deviation). Similar to the compressive strength results, the families without sodium silicate (i.e., N8S0, N10S0, N12S0, and N14S0) showed the best performances, except for a few cases that require further investigation. Again, the lowest values were seen for the alkali-activated binders with SiO_2_/Na_2_O ratio of 0.5. For the FA mixes, families N10S1.5, N10S1, and N12S1 showed top values of 8.6 MPa, 7.9 MPa, and 7.9 MPa, respectively (Figure 10b,c). For mixes with MIBA, EAFS, and WGR, the presence of sodium silicate in the alkaline solution reduced the performance. Furthermore, for the MIBA mixes, the maximum flexural strength values were achieved at N14S0, N10S0, and N12S0 with values of 3.4 MPa, 3.3 MPa, and 3.3 MPa, respectively (Figure 10b–d). In addition, for EAFS, the maximum values were achieved at N12S0 and N14S0 with 6.7 MPa and 6.5 MPa, respectively (Figure 10c,d). Moreover, in correlation with the results obtained from the compressive strength test performed on WGR mixes, the flexural strength’s maximum performance was achieved at WGR mixes N10S0, N12S0, and N8S0 with 6.0 MPa, 5.0 MPa, and 4.8 MPa, respectively.

**Figure 11:**
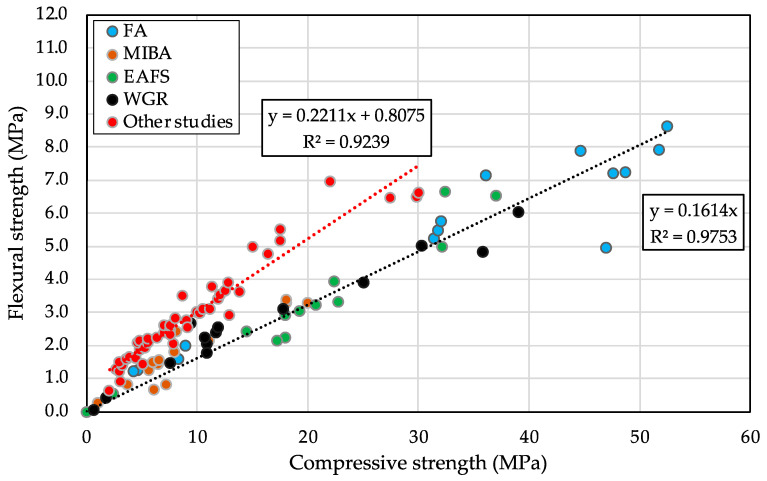
Flexural versus compressive strengths of all alkali-activated binders of this study compared to other studies from the literature [92,93,94,95,96,97,98,99].

A correlation between the flexural strength results and the compressive strength ones was established (Figure 11). Based on the trendline, the positive correlation coefficient close to 1 (R^2^ ≈ 0.98) indicated that the two variables (flexural (*y*-axis) and compressive (*x*-axis) strengths) are highly correlated, regardless of the variating ingredients’ concentrations of the alkaline activator and the different binders studied. This relationship seemed to be slightly affected by these mentioned varying factors. According to studies from the literature (Figure 11), a slightly lower correlation coefficient (R^2^ ≈ 0.92) was seen for mortars made with OPC. However, AAMs showed lower flexural strength values for given compressive strengths, compared to mortars with OPC as a binder [92].

Similar to the compressive strength results, FA had the best flexural strength performance, followed by WGR, which showed competitive results for mixes with no sodium silicate content in the alkaline solution. The EAFS’s mechanical performance comes behind WGR, also without the need for sodium silicate. Finally, the MIBA’s highest-performing mixes came in the final place, with also no presence of sodium silicate in the alkaline solution. From an economic point of view, it is worth noting that WGR showed the highest performance when compared to MIBA and EAFS with no sodium silicate and with relatively low sodium hydroxide contents (Na_2_O/binder ratios 8% and 10%, respectivley), while the MIBA and EAFS peaks were shown at higher sodium hydroxide contents (Na_2_O/binder ratios of 12% and 14%, respectively). Since the sodium silicate solution is considered the most expensive alkaline solution constituent, achieving the highest performance without the need for it can be economically feasible. In addition, the cost could be further reduced by decreasing the sodium hydroxide content in the alkaline solution, similar to the case of WGR. Moreover, the manufacturing process of sodium silicate solution generates CO_2_, thus more environmental benefits will be offered if it is not used.

## 4. Conclusions

The optimization of the alkaline solution for four different aluminosilicate industrial wastes was thoroughly investigated in this study, with the aim of improving their mechanical performances. The fresh and hardened state performance evaluations have the following conclusions: (i) From the four binders studied, fly ash, used as a reference precursor for alkali-activated binders, outperformed all other binders with respect to strength development. The fly ash specimens showed a denser microstructure that was even further densified with the exposure to CO_2_ curing. Unlike the municipal solid waste incineration bottom ashes, electric arc furnace slag, and waste glass rejects, which showed peak performance in alkaline solutions with common concentrations, fly ash showed its peak at different families, signifying the presence of distinct chemical reactions taking place; (ii) The waste glass rejects seconded, showing reasonably acceptable performance, followed by slag with comparable results, and finally, municipal waste incineration ashes that showed the lowest performance; (iii) The fly ash top-performing mixes were those with Na_2_O/binder ratio of 10 and SiO_2_/Na_2_O ratios of 1 and 1.5 (N10S1 and N10S1.5), after presenting high workability that helped create well-compacted specimens with a dense microstructure; (iv) The municipal solid waste incineration ashes, slag, and glass rejects mixes with the best performance were those with Na_2_O/binder ratios of 8, 10, 12, and 14 and with no sodium silicate content (N8S0, N10S0, N12S0, and N14S0, respectively); (v) The lowest performances were recorded for mixes with SiO_2_/Na_2_O ratio of 0.5 (N8S0.5, N10S0.5, N12S0.5, and N14S0.5) regardless of the precursor, after presenting flash setting, which resulted in dry and hard to compact mixes; (vi) Increasing the Na_2_O/binder ratio improved the overall performance; however, it decreased the performance of WGR-containing mixes. This could be related to the high amount of sodium hydroxide required to activate the precursor, given that a considerable amount of Na^+^ could be sourced from the binder itself. The WGR mixes with Na_2_O/binder ratios of 12 and 14 and no sodium silicate content (N12S0 and N14S0) presented efflorescence caused by the precipitation of sodium carbonates on the surface; (vii) WGR showed the highest flexural and compressive strength results at the lowest sodium hydroxide content and without the need for sodium silicate, which can reduce the cost and the dependence on these chemicals. The slag and municipal waste incineration ashes also showed their best performances without the need for sodium silicate; however, the sodium hydroxide content was the highest among the studied.

The newly investigated binders studied in this paper present adequate properties when compared to corresponding fly ash and ordinary Portland cement specimens. Therefore, given the considerable potential demonstrated here, additional investigation is needed on the alkali activation of the aforementioned waste aluminosilicate precursors to further improve their properties as construction materials.

## Figures and Tables

**Table 1:** Denomination of the alkaline solution of alkali-activated mixes.

	Na_2_O/Binder (%)	8	10	12	14
SiO_2_/Na_2_O					
0		N8S0	N10S0	N12S0	N14S0
0.5		N8S0.5	N10S0.5	N12S0.5	N14S0.5
1.0		N8S1.0	N10S1.0	N12S1.0	N14S1.0
1.5		N8S1.5	N10S1.5	N12S1.5	N14S1.5

Note: “N” represents the Na_2_O/binder ratio and “S” the SiO_2_/Na_2_O ratio.

**Table 2:** Mix composition for all binders.

Type of Binder	Mix Code	Binder	WRA	Water	Fine Sand	Coarse Sand	Sand Gravel	NaOH	Na_2_SiO_3_ Solution	Borax
OPC	-	350	5.3	136.1	300.3	581.3	1035.3	-	-	-
AAMs	N8S0	350	5.3	135.2	300.3	581.3	1035.3	36.1	0.0	14
	N8S0.5	350	5.3	100.6	300.3	581.3	1035.3	30.4	53.0	14
	N8S1.0	350	5.3	66.0	300.3	581.3	1035.3	24.6	106.1	14
	N8S1.5	350	5.3	31.4	300.3	581.3	1035.3	18.9	159.1	14
	N10S0	350	5.3	135.1	300.3	581.3	1035.3	45.5	0.0	14
	N10S0.5	350	5.3	91.9	300.3	581.3	1035.3	38.0	66.3	14
	N10S1.0	350	5.3	48.6	300.3	581.3	1035.3	30.8	132.6	14
	N10S1.5	350	5.3	5.4	300.3	581.3	1035.3	23.6	198.9	14
	N12S0	350	5.3	135.0	300.3	581.3	1035.3	54.5	0.0	14
	N12S0.5	350	5.3	83.1	300.3	581.3	1035.3	45.6	79.5	14
	N12S1.0	350	5.3	31.2	300.3	581.3	1035.3	37.0	159.1	14
	N14S0	350	5.3	135.0	300.3	581.3	1035.3	63.6	0.0	14
	N14S0.5	350	5.3	74.4	300.3	581.3	1035.3	53.2	92.8	14
	N14S1.0	350	5.3	13.8	300.3	581.3	1035.3	43.1	185.6	14

**Table 3:** Curing stages and conditions.

Binders	Stage 1	Stage 2	Stage 3
	24 h	21 Days	7 Days
OPC	Spray with water	Dry chamber (23 ± 2 °C and 65% RH). Sprayed with water twice a day for the first 2 days	Carbonation chamber (23 ± 2 °C, 65% RH, and 5% CO_2_)
FA, MIBA, EAFS, and WGR	Thermal curing (70 °C)	Dry chamber (23 ± 2 °C and 65% RH)	Carbonation chamber (23 ± 2 °C, 65% RH, and 5% CO_2_)

**Table 4:** Chemical composition of OPC, FA, MIBA, EAFS, and WGR obtained from XRF (%).

Materials	OPC (%)	FA (%)	MIBA (%)	EAFS (%)	WGR (%)
Al_2_O_3_	5.42	25.5	8.82	10.2	1.01
CaO	64.8	2.27	18.3	28.2	8.74
Fe_2_O_3_	2.92	6.90	6.68	28.5	0.67
K_2_O	0.74	2.74	1.59	0.03	0.40
MgO	2.12	1.83	4.01	5.67	3.55
Na_2_O	0.14	1.29	6.53	0.18	11.8
SiO_2_	18.1	56.3	48.8	17.7	71.4
SO_3_	4.81	0.80	1.36	0.33	0.30
Cl^-^	0.00	0.00	0.00	0.00	0.00
Cr_2_O_3_	0.51	0.48	0.06	2.38	0.03
TiO_2_	0.34	1.14	0.48	0.65	0.05
ZnO	-	-	-	-	-
P_2_O_5_	0.03	0.44	2.51	0.42	-
V_2_O_5_	0.02	0.05	-	0.11	-
CuO	-	-	-	-	0.02
MnO_2_	-	-	0.12	5.45	-

**Table 5:** Flow table results of FA, MIBA, EAFS, and WGR.

Combination	FA	MIBA	EAFS	WGR
	(mm)	(mm)	(mm)	(mm)
N8S0	125.4	–	–	104.7
N8S0.5	101.4	–	103.2	102.5
N8S1.0	131.8	103.7	107.8	103.2
N8S1.5	128.8	103.2	104.7	104.6
N10S0	122.4	104.1	–	105.6
N10S0.5	–	–	–	–
N10S1.0	135.8	106.1	102.6	105.7
N10S1.5	122.5	103.0	102.7	102.1
N12S0	128.5	105.8	104.7	127.4
N12S0.5	–	–	–	–
N12S1.0	124.0	104.2	103.1	102.2
N14S0	131.3	102.3	103.8	150.9
N14S0.5	–	–	–	–
N14S1.0	126.5	103.3	103.4	103.1

Note: “–” means that the value was equal to 100 mm and thus inconclusive.

## Data Availability

The data presented in this study are available on request from the corresponding author.
